# Joint physical-activity/screen-time trajectories during early childhood: socio-demographic predictors and consequences on health-related quality-of-life and socio-emotional outcomes

**DOI:** 10.1186/s12966-019-0816-3

**Published:** 2019-07-08

**Authors:** Borja del Pozo-Cruz, Francisco Perales, Phil Parker, Chris Lonsdale, Michael Noetel, Kylie D. Hesketh, Taren Sanders

**Affiliations:** 1 Motivation and Behaviour Research Program, Institute for Positive Psychology and Education, Faculty of Health Sciences, Australian Catholic University, Street: Level 10, 33 Berry Street, North Sydney NSW 2060, PO Box 968, Sydney, 2059 Australia; 2 ARC Centre of Excellence for Children and Families over the Life Course (Life Course Centre), Institute for Social Science Research, The University of Queensland, Brisbane, Australia; 3 Institute for Physical Activity and Nutrition, School of Exercise and Nutrition Science, Faculty of Health, Deakin University, Geelong, Australia

**Keywords:** Time use, Trajectory analysis, Physical activity, Screen time, Child development, Health

## Abstract

**Background:**

Understanding the early roots of physical activity and sedentary behaviors is critical to developing intervention programs that promote healthy lifestyle habits in infants and children. There is, however, no evidence on how these behaviors cluster and develop together during early childhood. The aim of this study was to identify single and joint longitudinal trajectories in physical activity and screen time amongst children aged 0 to 9 years, their social-demographic predictors and their prospective health-related quality-of-life and socio-emotional outcomes.

**Methods:**

Three waves of data from *The Longitudinal Study of Australian Children*, a national study tracking two cohorts every 2 years (B-cohort, 0–5 years, *n* = 4,164; K-cohort, 4–9 years, *n* = 3,974) were analysed. Growth mixture modelling was applied to longitudinal time-use diary data to identify joint trajectories in children’s physical activity and screen time over Waves 1–3. Key socio-demographic variables measured at Wave 1 were used to predict membership in different trajectories*.* The prospective consequences (at Wave 3) of time-use trajectories on health-related quality-of-life and socio-emotional outcomes were assessed.

**Results:**

Three physical-activity/screen-time trajectories were identified for both cohorts: Cluster-A—children who maintained low levels of physical activity and screen time (*∽50% of the sample),* Cluster-B—children who progressively increased physical activity and maintained low screen-time levels (*∽25%*), and Cluster-C—children who maintained low physical-activity levels and increased screen time (*∽25%*). Children in Cluster-B experienced the best health-related quality-of-life and socio-emotional outcomes, while those in Cluster-C experienced the worst. Children who were female, Indigenous, from non-English-speaking backgrounds, not living with two biological parents, in more affluent households and neighbourhoods, without siblings and with parents with poor mental health were at greater risk of falling into Cluster-A or Cluster-C.

**Conclusion:**

Our findings identified which children are most at-risk of falling into time-use trajectories that lead to poor health-related quality-of-life and socio-emotional outcomes later in life, increasing our ability to monitor, detect and prevent these suboptimal behaviours prior to their onset.

**Electronic supplementary material:**

The online version of this article (10.1186/s12966-019-0816-3) contains supplementary material, which is available to authorized users.

## Background

Increasing physical activity and reducing sedentary behaviors improves children’s health and wellbeing [1–5]. For instance, recent systematic reviews conclude that physical activity is positively associated with a broad range of psychological, cognitive, and cardio-metabolic child outcomes, while sedentary behaviours are negatively associated with these outcomes [6–9]. However, most existing studies are based on cross-sectional data [8, 9], which precludes examination of within-individual changes over time. Researchers are beginning to examine childhood screen-time and physical-activity behaviors as longitudinal trajectories [10–14]. These designs are unique in that they make the most of prospective datasets to generate novel insights on developmental dynamics; they allow prediction, with some degree of confidence, of the number and characteristics of children who will experience trajectories towards healthier or unhealthier behaviors over their childhood. This knowledge is critical to devising targeted and efficient early intervention programs aimed at developing healthy lifestyle habits from the first years of life. Intervening in the earliest years of life shifts the focus from remedial to preventive strategies, and reduces the burden on the public health system.

To date, this body of research [12–14] includes largely studies examining physical-activity trajectories [10–14], with a smaller pool of studies also considering TV-viewing [10] and sedentary-behavior [11] trajectories. For example, a study of 438 children aged 0–5 years in New Zealand identified four prevailing physical-activity trajectories (constantly low, increasing, decreasing and constantly high) and a similar set of trajectories for sedentary behaviors [11]. These trajectories were significantly associated with subsequent adiposity: children who maintained high levels of physical activity since birth had less fat mass by age 5 than children who experienced declining or stable physical-activity levels [11].

Despite its significant value, this pioneering research [10–14] remains limited in several ways. First, it treats sedentary behaviors (including screen time) and physical activity as separate constructs, not recognising that time spent in one domain might be intertwined with time spent in the other via potential substitution effects and latent lifestyle choices [15]. Second, it does not identify the characteristics of children who fall into different developmental trajectories (e.g., their gender, ethnicity, or socio-economic background). Third, it does not consider the consequences of trajectories on broader childhood physical and mental health outcomes (only adiposity). Finally, it relies on small, community, or non-probability samples.

In this study, we analyse longitudinal time-use diary data from two national cohorts of Australian children aged 0–5 and 4–9 years using state-of-the-art non-parametric growth mixture models. To our knowledge, we are the first to describe single and jointly-determined trajectories in screen time and physical activity over early childhood, paying attention to how these behaviors cluster and develop together. We also believe that we are also the first to examine the socio-demographic factors predicting membership in different time-use trajectories, and assess their consequences on health-related quality-of-life (HRQoL) and socio-emotional outcomes. Collectively, these analyses yield unique and important insights about which children are most at-risk of falling into time-use trajectories that lead to poor HRQoL and socio-emotional outcomes later in life, increasing our ability to monitor, detect, and prevent these suboptimal behaviours prior to their onset.

## Method

### Dataset

We used data from *The Longitudinal Study of Australian Children* (LSAC), a population-based study which tracks every 2 years two cohorts of children aged 0/1 years (B-cohort) and 4/5 years (K-cohort) at inception in 2004. The data were collected using a complex, probabilistic methodology that yielded a highly-representative sample of Australian children of those ages. The analyses were based on data from the first three LSAC waves for the B-cohort (ages 0/1, 2/3 & 4/5) and the K-cohort (ages 4/5, 6/7 & 8/9). Data from subsequent waves could not be included due to significant changes in the design of the time-use diary instrument [16]. The initial study wave accomplished an overall response rate of 67% in the B-cohort and 62% in the K-cohort, with Wave 3 retention rates of 86% for the B-cohort and 87% for the K-cohort. Further details on the LSAC methodology have been published elsewhere [17].

### Children’s time use

The measures of children’s physical activity and screen time were derived from LSAC’s time-use diaries. This instrument was completed by a child’s guardian for one weekday and one weekend day. All child activities were recorded every 15 min for all waking hours using pre-determined codes [16]. The time-use codes allocated to physical activity and screen time for each wave and cohort are shown in Online Additional file 1. Missing data on child activities in each of the 96 time blocks was dealt with through a two-step procedure. First, we calculated the modal activity in each 15-min time block across subgroups defined by cohort (B/K), wave (1/2/3), day type (weekday/weekend), and day-time (each 15-min block). We then use the calculated modes to impute observations with missing data for children in each subgroup. For example, if a child had missing data on the activity he/she conducted between 10 am and 10.15 am on a weekday, the most frequent activity undertaken on that time and day by other children of the same cohort and age would be imputed. Weekly measures of children’s physical activity and screen time were constructed as follows: (weekday total*5) + (weekend total*2). To prevent over-influential outliers from affecting the analyses, values in the time-use variables that were greater than the 99th percentile of the variable distribution were top-coded (i.e., they were substituted with the value of the 99th percentile). Time-use diary data were available for 67% (*n* = 18,643) of the observations in LSAC waves 1–3. We subsequently excluded cases with missing information on the day of the week in which the diary was completed (*n* = 43), with two weekdays or two weekend days instead of one of each (*n* = 311), and with missing information on either of the two diaries (*n* = 224). This resulted in an unbalanced sample comprising 17,406 observations from 8,143 children distributed as follows: 9,263 observations from 4,164 B-cohort children (Wave 1 = 3,454; Wave 2 = 3,072; Wave 3 = 2,737) and 8,802 observations from 3,979 K-cohort children (Wave 1 = 3,149; Wave 2 = 2,984; Wave 3 = 2,669).

We computed time-use trajectories separately for children in the B- and K–cohorts, given their different longitudinal age profiles. To do so, we first dealt with missing data in the time-use variables due to wave non-response through the copy mean imputation approach, a two-step procedure in which linear interpolation based on existing data is first used to impute a value and then the value is shrunk toward the average trajectory [18]. Therefore, full trajectories were retrieved for the 4,164 B-cohort and 3,979 K-cohort children observed at least once. Non-parametric, expectation-maximisation growth mixture modelling was then used to identify clusters of children experiencing similar single and joint physical activity and screen time trajectories. To extract the optimal number of clusters and ensure convergence, 20 iterations of the *K*-means algorithm were performed. The optimal number of clusters (i.e., trajectories) was determined using the Calinski/Harabatz criterion [19]. This involved calculating a ratio between measures of dissimilarity *between* and *within* clusters across solutions with different numbers of clusters. The solution with the highest value was then deemed optimal and used in the analyses [19].

### Socio-demographic predictors

Measured variables included child’s gender (male/female), age (in months), Indigenous background (yes/no), birth weight at or below 2.5 kg. (yes/no), area-level socio-economic background (*Socio-Economic Index for Areas* [20]), main carer’s mental health (*Kessler 6* scale [21]), main language spoken at home (English/other), weekly parental income (adjusted to 2008 prices using the Consumer Price Index), and whether the study child lived with siblings (yes/no) and two biological parents (yes/no) (for descriptive statistics, see Table 1). These predictors were selected due to their inclusion and predictive power in previous studies of screen time, physical activity, and/or child outcomes [22–25].

**Table 1:** Descriptive statistics on the study sample

	B-cohort (0–5 years)	K-cohort (4–9 years)
	Mean (SD) or %	Mean (SD) or %
*Time use (Waves 1 to 3)*		
Physical activity (hours/week)		
Wave 1	8.04 (7.84)	11.35 (6.87)
Wave 2	9.99 (7.04)	10.47 (7.14)
Wave 3	9.53 (7.15)	11.20 (8.13)
Screen time (hours/week)		
Wave 1	2.05 (3.19)	11.71 (7.44)
Wave 2	8.78 (5.97)	10.76 (7.18)
Wave 3	10.40 (7.04)	13.25 (8.66)
*Child outcomes (Wave 3)*		
Health-related quality-of-life (PedsQL)		
Total score (0–100)	82.48 (9.66)	80.60 (11.81)
Physical health (0–100)	84.78 (10.45)	84.06 (13.76)
Social health (0–100)	84.30 (14.20)	80.00 (17.14)
Emotional health (0–100)	74.62 (13.91)	73.37 (15.54)
Socio-emotional outcomes (SDQ)		
Total score (0–40)	7.92 (4.56)	7.08 (5.10)
Hyperactivity (0–10)	3.16 (2.04)	3.05 (2.31)
Peer problems (0–10)	1.33 (1.46)	1.37 (1.60)
Conduct problems (0–10)	2.05 (1.74)	1.22 (1.40)
Emotional problems (0–10)	1.38 (1.45)	1.45 (1.66)
*Predictors/covariates (Wave 1)*		
Female, *yes, %*	48.41	47.76
Indigenous, *yes, %*	2.61	2.13
SEIFA (0–14)	10.08 (0.80)	10.10 (0.79)
Low birth weight, *yes, %*	5.32	6.42
Main caregiver mental health (0–5)	4.44 (0.55)	4.50 (4.43)
English as first language, *yes, %*	92.32	90.79
Siblings, *yes, %*	22.18	38.33
Two biological parents, *yes, %*	92.94	87.42
Parental income (AU$ per week)	1,307.11 (1,029.13)	1,387.06 (1,068.16)

LSAC data, Waves 1–3. *PedsQl* Pediatric Quality of Life inventory, *SDQ* Strengths and Difficulties Questionnaire, *SEIFA* Socio-Economic Index for Areas

### Child outcomes

Children’s health-related quality-of-life (HRQoL) was assessed via the *Paediatric Quality of Life Inventory* (PedsQL), a 23-item parent-reported instrument [26]. This instrument has demonstrated reliability, validity, sensitivity and responsiveness for parental reports of children ages 2–18 years, and is related in meaningful ways to key constructs in paediatric healthcare [27]. The analyses considered the PedsQL Total Score and three subscales (physical, social, and emotional functioning), all of which ranged from 0 to 100. Higher PedsQL scores denote better HRQoL in children. Children’s socio-emotional outcomes were assessed via the *Strengths and Difficulties Questionnaire* (SDQ), a 25-item parent-reported instrument [28]. This measure has demonstrated sound psychometric properties—such as reliability and validity—among Australian children [29]. Both the SDQ Total Score (range: 0–40) and its four subscales (conduct problems, emotional problems, hyperactivity/inattention and peer problems; range: 0–10) were used. Higher SDQ scores denote more socio-emotional problems.

### Estimation strategy

Multinomial logistic regression models were fitted to examine how socio-demographic factors measured at Wave 1 predicted membership in different physical-activity/screen-time trajectories over Waves 1–3. Their results are reported as odds ratios (ORs) and their magnitude illustrated through average marginal effects (AMEs) [30]. Ordinary least squares regression models were used to examine the associations between membership in different physical-activity/screen-time trajectories over Waves 1–3 and children’s HRQoL and social-emotional outcomes at Wave 3. These models were adjusted for the socio-demographic factors measured at Wave 1 to reduce the risk of confounding. We report both unstandardized and standardized beta coefficients for these models. The latter are informative of the effect sizes (ES) [31]. The estimation samples range from 2,062 to 2,219 children, depending on missing data in the control and outcome variables used (see Online Additional file 2). All regression models were estimated separately for the B- and K-cohorts and conducted using *R* software version 3.4.4 [32]. A range of sensitivity analyses was performed to test the robustness of the findings to different methodological decisions with satisfactory results (see Online Additional file 3).

## Results

### Time-use trajectories

**Fig. 1:**
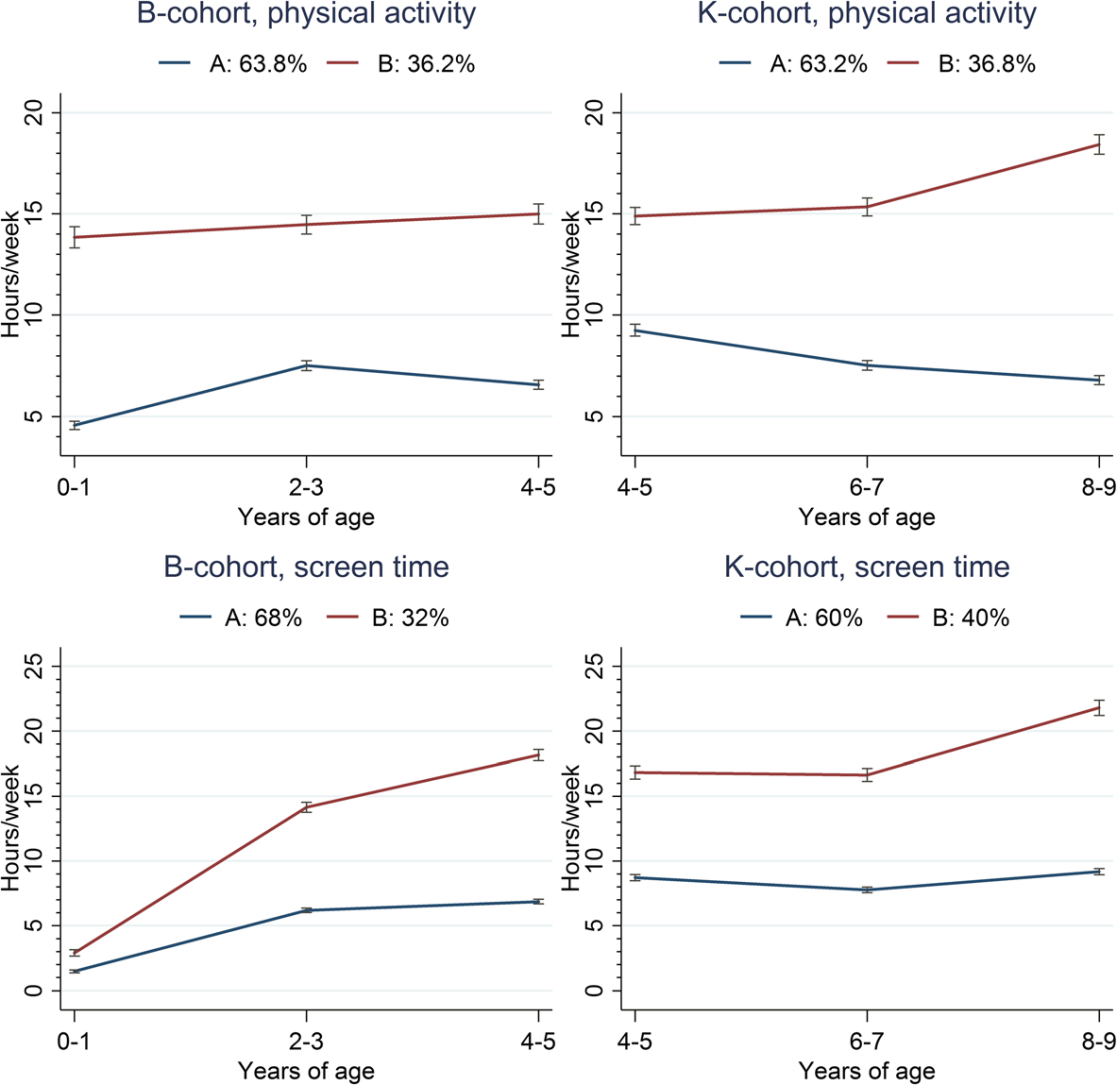
Single physical activity and screen time for B and K cohorts

Figure 1 displays the identified *single* physical-activity and screen-time trajectories. For B-cohort children, a two-cluster solution for physical-activity trajectories (Cluster-A: *consistently low activity*, 63.8% of children; Cluster-B: *consistently high activity*, 36.2% of children) and a two-cluster solution for screen-time trajectories (Cluster-A: *moderate-increasers*; Cluster-B: 68%; *rapid increasers*, 32%) were identified (Fig. 1). For K-cohort children, two-cluster solutions were identified for trajectories in both physical activity (Cluster-A: *steady decliners*, 63.2%; Cluster-B: *late increasers*, 36.8%) and screen time (Cluster-A: *maintainers*, 60%; Cluster-B: *late increasers*, 40%) (Fig. 1).

**Fig. 2:**
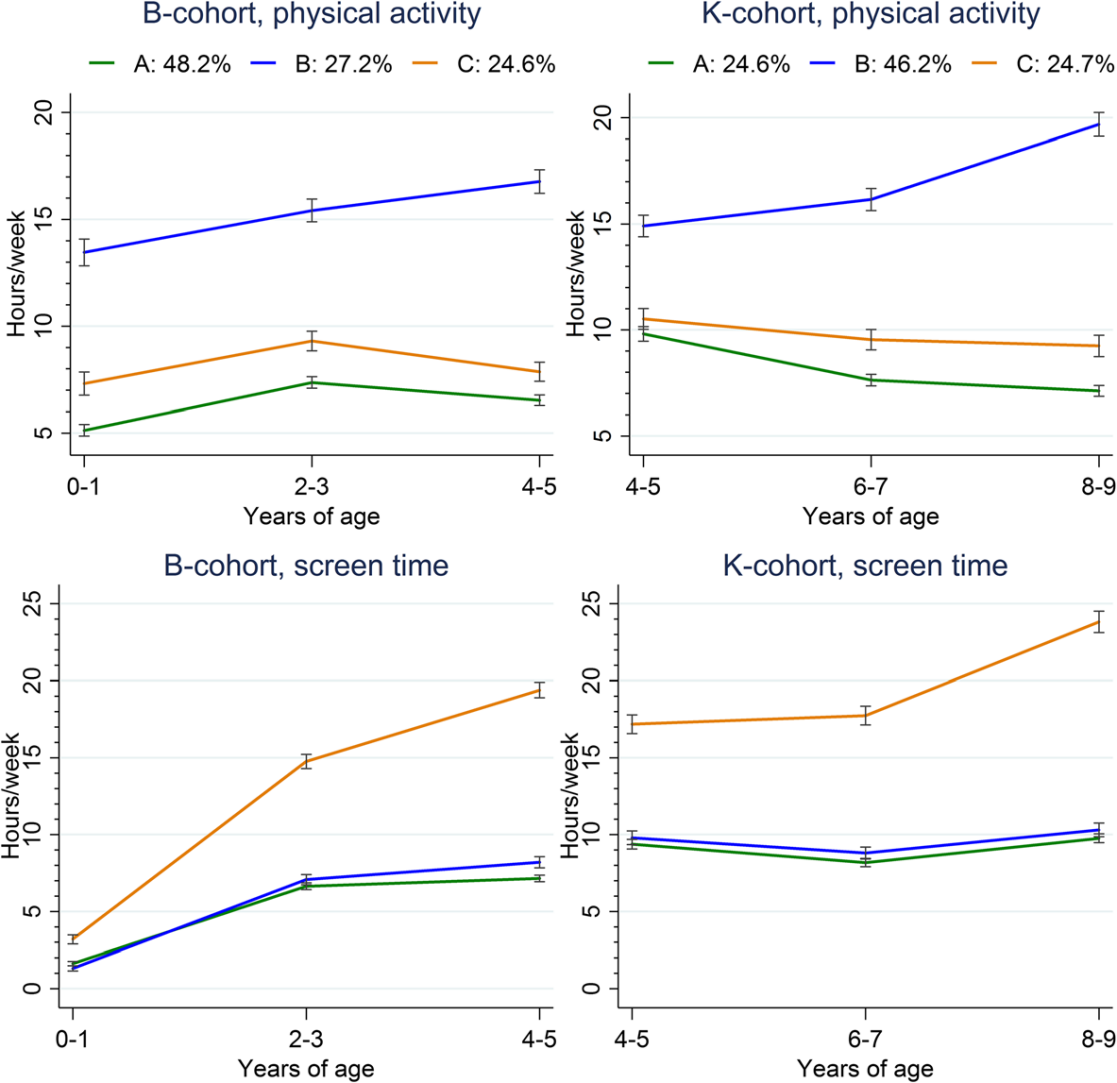
Joint physical activity and screen time for B and K cohorts

Figure 2 displays the identified *joint* physical-activity/screen-time trajectories. A three-cluster solution for joint physical-activity/screen-time trajectories was identified for B-cohort children. The first cluster comprised children who maintained low levels of physical activity and screen time over time (Cluster-A: *low activity-low screen*, 48.2%), the second comprised children who progressively increased their physical activity levels and maintained low levels of screen time (Cluster-B: *increasing activity-low screen*, 27.2%), and the third comprised children who maintained low levels of physical activity and steadily increased their screen time (Cluster-C: *low activity-increasing screen*, 24.6%). Similar clusters were identified for K-cohort children (Cluster-A: *low activity-low screen*, 46.2%; Cluster-B: *increasing activity-low screen*, 29.1%; Cluster-C: *low activity- increasing screen*, 24.7%) (Fig. 2).

### Predictors of physical-activity/screen-time trajectories

(ORs in Table 2; AMEs in Online Additional file 4)*.* In the B-cohort, compared to children in the *increasing activity-low screen* category, those in the *low activity-low screen* category were significantly more likely to be female (OR = 1.701, *p* < 0.001; AME = 0.135, *p* < 0.001), have no siblings (OR = 0.647, *p* < 0.001; AME = ─0.076, *p* < 0.001), not live with two biological parents (OR = 0.557, *p* < 0.01; AME = ─0.101, *p* < 0.05), live in a high socio-economic status area (OR = 1.268, *p* < 0.001; AME = 0.056, *p* < 0.001), have parents with high incomes (OR = 1.143, *p* < 0.001; AME = 0.036, *p* < 0.001), and have parents with poorer mental health (OR = 0.792, *p* < 0.05; AME = ─0.039, *p* < 0.05). Compared to children in the *increasing activity-low screen* category, those in the *low activity-increasing screen* category were significantly more likely to have no siblings (OR = 0.775, *p* < 0.05; AME = 0.001, *p* > 0.05) and speak English as a second language (OR = 0.603, *p* < 0.05; AME = ─0.069, *p* < 0.05).

**Table 2:** Predictors of membership in different physical-activity/screen-time trajectory groups from multinomial logistic regression models

	B-cohort (0–5 years) (reference: *Increasing activity-low screen*)						K-cohort (4–9 years) (reference: *Increasing activity-low screen*)					
	*Low activity-* *low screen*			*Low activity-* *increasing screen*			*Low activity-* *low screen*			*Low activity-* *increasing screen*		
	*OR*	*95% CI*	*p*	*OR*	*95% CI*	*p*	*OR*	*95% CI*	*p*	*OR*	*95% CI*	*p*
Female, *yes*	1.701	1.418–2.039	<.001	0.949	0.770–1.170	.624	2.027	1.678–2.448	<.001	0.913	0.736–1.134	.411
Indigenous, *yes*	1.042	0.514–2.110	.909	1.863	0.923–3.759	.082	0.862	0.386–1.926	.718	1.441	0.650,3.196	.368
SEIFA (0–14)	1.268	1.126–1.428	<.001	1.005	0.875–1.155	.939	1.058	0.936–1.196	.369	0.871	0.756–1.004	.057
Low birth weight, *yes*	1.327	0.856–2.057	.206	1.033	0.617–1.730	.902	0.814	0.552–1.200	.298	0.775	0.496–1.209	.261
Main caregiver mental health (0–5)	0.792	0.663–0.946	.010	0.865	0.706–1.061	.165	0.868	0.727–1.035	.114	0.763	0.630–0.923	.005
English as first language, *yes*	0.786	0.533–1.160	.226	0.603	0.396–0.918	.018	0.501	0.341–0.734	<.001	0.692	0.445–1.077	.103
Siblings, *yes*	0.647	0.521–0.804	<.001	0.775	0.606–0.992	.043	0.743	0.612–0.902	.003	0.804	0.646–1.001	.051
Two biological parents, *yes*	0.557	0.364–0.853	.007	0.727	0.450–1.173	.191	0.663	0.469–0.937	.020	0.799	0.545–1.173	.252
Weekly parental income (in AU$1,000)	1.143	1.039–1.257	.006	0.962	0.856–1.080	.511	1.029	0.939–1.128	.536	0.984	0.883–1.096	.773
Cases	2797							2551				
Pseudo R^2^	0.30							0.36				

LSAC data, waves 1–3. *OR* Odd ratios. *95% CI* 95% Confidence interval, *SEIFA* Socio-Economic Index for Areas

In the K-cohort, compared to children in the *increasing activity-low screen* category, children in the *low activity-low screen* category were significantly more likely to be female (OR = 2.027, *p* < 0.001; AME = 0.181, *p* < 0.001), have no siblings (OR = 0.743, *p* < 0.01; AME = ─0.047, *p* < 0.05), speak English as a second language (OR = 0.501, *p* < 0.001; AME = ─0.121, *p* < 0.001), and not live with two biological parents (OR = 0.663, *p* < 0.05; AME==─0.072, *p* < 0.05). Compared to children in the *increasing activity-low screen* category, those in the *low activity-increasing screen* category were significantly more likely to have main caregivers with poorer mental health (OR = 0.763, *p <* 0.01; AME = ─0.035, *p* < 0.05).

### Physical-activity/screen-time trajectories as predictors of children’s HRQoL

(Table 3)*.* In the B-cohort, compared to children in the *increasing activity-low screen* category, those in the *low activity-low screen* and *low activity-increasing screen* categories had lower PedsQL total scores (*β =* ─0.96, ES = ─0.10, *p =* 0.047 & *β =* ─1.44, ES = ─0.15, *p =* 0.010) and lower PedsQL physical health scores (*β =* ─1.60, ES = ─0.015, *p =* 0.002 & *β =* ─2.00, ES = ─0.19, *p* = 0.001) at Wave 3.

**Table 3:** Associations between joint physical-activity/screen-time trajectories and health-related quality-of-life outcomes (PedsQL)

	PedsQL - total score				PedsQL- physical health				PedsQL- social health				PedsQL- emotional health			
	*β*	*95% CI*	*ES*	*p*	*β*	*95% CI*	*ES*	*p*	*β*	*95% CI*	*ES*	*p*	*β*	*95% CI*	*ES*	*p*
B-cohort (0–5 years)																
Joint trajectories (Reference: *Increasing activity-low screen*)																
*Low activity-low screen*	−0.96	−1.92 – − 0.01	− 0.10	.047	−1.60	−2.63 – − 0.56	− 0.15	**.002**	− 0.67	−2.08 – 0.73	− 0.05	.349	− 0.61	−1.97 – 0.76	− 0.04	.385
*Low activity-increasing screen*	−1.44	−2.54 – − 0.34	− 0.15	.010	−2.00	−3.20 – − 0.81	− 0.19	**.001**	− 0.60	− 2.22 – 1.03	− 0.04	.473	−1.22	− 2.80 – 0.36	−0.09	.131
Cases	2215				2216				2207				2214			
R^2^ / adjusted R^2^	.060 / .056				.041/ .056				.039 / .034				.067/ .063			
K K-cohort (4–9 years)																
Joint trajectories (Reference: *Increasing activity-low screen*)																
*Low activity-low screen*	−1.40	−2.55 – −0.25	−0.12	**.017**	−1.50	−2.84 – − 0.15	−0.11	**.029**	− 1.95	−3.64 – − 0.25	−0.12	**.024**	−0.84	−2.39 – 0.71	− 0.05	.288
*Low activity-increasing screen*	−2.29	−3.61 – −0.97	− 0.20	**.001**	− 3.19^a^	−4.73 – − 1.66	−0.24	**.001**	−2.19	−4.13 – − 0.25	−0.13	**.027**	−0.58	−2.35 – 1.20	− 0.04	.523
Cases	2062				2062				2062				2062			
R^2^ / adjusted R^2^	0.101 / 0.096				0.061 / 0.055				0.065 / 0.060				0.087 / 0.083			

LSAC data, Waves 1–3. *PedsQL* Paediatric Quality of Life Inventory. Unstandardized coefficients from ordinary least square regression models. All models control for child’s gender (male/female), age (in months), Indigenous background (yes/no), birth weight at or below 2.5 kg. (yes/no), and area-level socio-economic background (measured by the Socio-Economic Index for Areas), main caregiver’s mental health (measured by the Kessler 6 scale), main language spoken at home (English/other), weekly parental income (adjusted to 2008 prices using the Consumer Price Index), and whether the study child had siblings (yes/no) and two biological parents (yes/no) in the household. ^a^ indicates statistically significantly different from coefficient on *low activity-low screen* (*p* < 0.05) in a Wald test. *ES* Effect size, calculated as *β* × (SD_x_/SD_y_)

Bold data is statistically significant

In the K-cohort, compared to children in the *increasing activity-low screen* category, those in the *low activity-low screen* and *low activity-increasing screen* categories had significantly lower PedsQL total scores (*β =* ─1.40, ES = ─0.12, *p =* 0.017 & *β =* ─2.29, ES = ─0.20, *p* = 0.001), PedsQL physical health scores (*β =* ─1.50, ES = ─0.11, *p =* 0.029 & *β =* ─3.19, ES = -0.24, *p* = 0.001) and PedsQL social health scores (*β =* ─1.95, ES = ─0.12, *p =* 0.024 & *β =* ─2.19, ES = ─0.13, *p =* 0.027) at Wave 3. Results from Wald tests indicated that K-cohort children in the *low activity-low screen* category had significantly better PedsQL physical health scores than those in the *low activity-increasing screen* category (*p* < 0.05).

### Physical-activity/screen-time trajectories as predictors of children’s socio-emotional outcomes

(Table 4)*.* In the B-cohort, compared to children in the *increasing activity-low screen* category, those in the *low activity-increasing screen* category had significantly higher total SDQ scores (*β =* 0.69, ES = 0.15, *p* = 0.07) and SDQ hyperactivity scores (*β =* 0.36, ES = 0.18, *p* = 0.02). Results from Wald tests indicated that B-cohort children in the *low activity-low screen* category displayed significantly better scores than those in the *low activity-increasing screen* category in all outcomes (*p* < 0.05) except SDQ emotional problems (*p* < 0.1).

**Table 4:** Associations between joint physical-activity/screen-time trajectories and socio-emotional outcomes (SDQ)

	SDQ - total score				SDQ - hyperactivity				SDQ - peer problems				SDQ - conduct problems				SDQ - emotional problems			
	*β*	*95% CI*	*ES*	*p*	*β*	*95% CI*	*ES*	*p*	*β*	*95% CI*	*ES*	*p*	*β*	*95% CI*	*ES*	*p*	*β*	*95% CI*	*ES*	*p*
B-cohort (0–5 years)																				
Joint trajectories (Reference: *Increasing activity-low screen*)																				
*Low activity-* *low screen*	−0.22	−0.65 – 0.21	−0.05	.313	−0.03	− 0.23 – 0.17	−0.01	.776	−0.08	− 0.22 – 0.06	−0.05	.290	−0.06	− 0.23 – 0.11	−0.03	.503	−0.06	− 0.21 – 0.08	−0.04	.388
*Low activity-* *increasing screen*	0.69^a^	0.19–1.19	0.15	**.007**	0.36^a^	0.13–0.59	0.18	**.002**	0.13^a^	−0.03 – 0.29	0.09	.112	0.13^a^	−0.07 – 0.33	0.08	.193	0.07^b^	−0.10 – 0.23	0.05	.430
Cases	2218				2218				2219				2219				2219			
R^2^ / adjusted R^2^	.103 / .098				.076 / 0.071				.055 / 0.050				.054 / 0.050				.041 / 0.036			
B K-cohort (4–9 years)																				
Joint trajectories (Reference: *Increasing activity-low screen*)																				
*Low activity-* *low screen*	0.46	−0.03 – 0.94	0.09	.068	0.07	− 0.16 – 0.29	0.03	.570	0.24	0.09–0.40	0.16	**.002**	0.00	− 0.14 – 0.14	0.00	.999	0.15	− 0.02 – 0.32	0.09	.086
*Low activity-* *increasing screen*	0.74	0.18–1.30	0.15	**.010**	0.24	− 0.02 – 0.50	0.10	.070	0.39^b^	0.21–0.57	0.25	**<.001**	− 0.03	− 0.19 – 0.13	−0.02	.702	0.14	− 0.05 – 0.33	0.08	.152
Cases	2062				2062				2062				2062				2063			
R^2^ / adjusted R^2^	.135 / .131				.111 / .106				.077 / .072				.065 / .060				.076 / .071			

LSAC data, Waves 1–3. *SDQ* Strength and Difficulties Questionnaire. Unstandardized coefficients from ordinary least square regression models. All models control for child’s gender (male/female), age (in months), Indigenous background (yes/no), birth weight at or below 2.5 kg. (yes/no), and area-level socio-economic background (measured by the Socio-Economic Index for Areas), main caregiver’s mental health (measured by the Kessler 6 scale), main language spoken at home (English/other), weekly parental income (adjusted to 2008 prices using the Consumer Price Index), and whether the study child has siblings (yes/no) and two biological parents (yes/no) in the household. ^a^ statistically significantly different from coefficient on *low activity-low screen* (*p* < 0.05) in a Wald test, or ^b^ trending towards being significantly different (*p* < 0.1). *ES* Effect size, calculated as *β* × (SD_x_/SD_y_)

Bold data is statistically significant

In the K-cohort, compared to children in the *increasing activity-low screen* category, those in the *low activity-increasing screen* category had significantly higher total SDQ scores (*β =* 0.74, ES = 0.15, *p* = 0.010) and more SDQ peer problems (*β =* 0.39, ES = 0.25, *p* < 0.001), whereas children in the *low activity-low screen* category had significantly more SDQ peer problems (*β =* 0.24, ES = 0.16, *p* = 0.002).

## Discussion

While researchers and policymakers recognize the importance of increasing physical activity and reducing screen time in infants and young children, few studies have leveraged the power of maturing longitudinal datasets to understand these behaviors as jointly-determined developmental trajectories. This study of two national cohorts of Australian children aged 0–5 (*n* = 4,164) and 4–9 years (*n* = 3,979) was, to our knowledge, the first to map joint trajectories in physical-activity and screen-time behaviors, identify their socio-demographic antecedents, and establish their consequences on children’s HRQoL and socio-emotional outcomes.

Consistent with previous studies, we generally observed overall declines in physical activity and increases in screen time from the age of 4 as children grew older [33, 34]. However, our trajectory analyses revealed that only about one quarter of all children demonstrate this suboptimal pattern. Another quarter experienced longitudinal time-use trajectories characterised by increasing levels of physical activity and consistently low levels of screen time. The largest group of children (about half of the sample) exhibited time-use profiles characterised by low levels of both screen time and physical activity—a pattern previously reported for children older than those in our sample [10]. These results suggests that physical-activity and screen-time behaviors are related in complex ways [10], with trade-offs between them and—potentially—with other uses of time not considered here (e.g., sleep or other sedentary behaviors) [35]. These complex time-substitution processes deserve further investigation. Altogether, this set of results warns against assuming homogeneity in the physical-activity and screen-time behaviors of different children as they grow older.

Consistent with findings of studies focusing on single outcomes or trajectories, children with *joint* time-use trajectories characterised by increasing levels of physical activity and low levels of screen time displayed the highest HRQoL and the best social-emotional outcomes. Conversely, children with trajectories characterized by low levels of physical activity and increasing levels of screen time displayed the least desirable outcomes. While these associations were more pronounced for physical health, they also manifested for social and emotional health. This pattern of results suggests a longitudinal dose-response association between engagement in healthy time-use behaviors and positive HRQoL and socio-emotional outcomes in children from the early years. This aligns with current policy guidelines that emphasize the importance of establishing healthy lifestyle habits from birth [36–38]. Inspection of covariate-adjusted effect sizes—as reflected by standardized beta coefficients—revealed that these are of a small-to-moderate magnitude. For example, for the PedsQL, these ranged from 0.10 to 0.19 in the B-cohort and 0.11 to 0.24 in the K-cohort. For the SDQ, the analogous ranges of effect sizes were 0.15 to 0.18 in the B-cohort and 0.15 to 0.25 in the K-cohort. Continuous exposure to unhealthy time-use trajectories may exacerbate negative health-related outcomes as these individuals grow older [39]. Further, these effects also accumulate over the population, increasing the public health burden.

Our results also hint at the relative importance of physical-activity vs. screen-time trajectories in determining children’s HRQoL and socio-emotional outcomes. Differences in HRQoL outcomes between children experiencing *low activity-low screen* and *low activity-increasing screen* trajectories were rarely statistically significant (1 in 8 parameters; physical health in the K-cohort). In contrast, children in either of these trajectories often experienced significantly poorer HRQoL than children in the *increasing activity-low screen* trajectory (4 in 8 parameters for the B-cohort and 6 in 8 parameters for the K-cohort). This suggests that longitudinal changes in physical activity behaviors may be more important than analogous changes in screen-time behaviours in determining subsequent HRQoL [40]. For socio-emotional outcomes, however, the results for B-cohort children suggest that screen time matters more than physical activity—although the pattern is less clear for K-cohort children.

The preponderance of physical activity in explaining HRQoL may occur because the PedsQL measure incorporates physical health, and the connections between physical activity and physical health are well-established [6, 8]. In fact, the PedsQL physical-health subscale is the most strongly influenced by membership in the increasing physical-activity trajectory. The dominance of screen time in influencing socio-emotional outcomes may suggest that over-use of TVs, computers, and other screens may have behavioural and/or brain consequences, which may in turn manifest as internalising and externalising behaviours. This aligns with evidence suggesting that prolonged exposure to screen time is associated with poorer psychosocial outcomes amongst young children [41].

Concerning cohort differences, social health (PedsQL) and peer problems (SDQ) were negatively impacted by membership in either of the less healthy time-use trajectories in the K-cohort, but not the B-cohort. This may reflect the importance of physical activity for social interactions as children grow older (e.g., playing active games or engaging in team sports).

Given their important consequences on HRQoL and socio-emotional outcomes, identifying the socio-demographic factors predicting membership in different physical-activity/screen-time trajectories amongst infants and young children constitutes an important task; one that may contribute to developing timely and targeted interventions [42]. This study identified several factors associated with a lower likelihood of children falling into the healthiest time-use trajectory (*increasing activity-low screen*) and/or a higher likelihood of falling into the unhealthiest time-use trajectory (*low activity-low screen or low activity-increasing screen*). These factors included being female, not speaking English at home, not living with two biological parents, having no siblings, having a high household income, living in an advantaged neighbourhood, and having parents with poor mental health.

To gain insights into the magnitude of associations, we estimated AMEs. In the B-cohort, these suggested that the probability of membership into the healthiest time-use category increased by having two biological parents (an 8.7% increase), having siblings (7.5%), being male (6.4%), and speaking English as a first language (6.2%). The probability decreased by 3% for a one-unit increase in the SEIFA score, and increased by 3.9% for a one-unit increase in main caregiver mental health (*Kessler 6* scale). In the K-cohort, the probability increased by 10.6% for speaking English as a first language, 8.5% for being male, 6.6% for having two biological parents, and 5.5% for having siblings. Altogether, these effects appear to be of a small-to-moderate size. Further, the models’ pseudo-R^2^ values (0.30 for the B-cohort and 0.36 for the K-cohort) suggest that unobserved factors are responsible for a large share of children’s propensities to fall into different time-use trajectories.

Generally, the factors found to predict membership in unhealthy time-use categories align with those previously reported to reduce physical activity and/or increase screen time in point-in-time studies, and many are commonly identified risk factors for negative outcomes in children [43–45]. As an exception, B-cohort children who lived in more advantaged households (as reflected by parental income) or neighbourhoods (as reflected by SEIFA scores) were more likely to fall into the *low activity-low screen* time-use category than into the healthier *increasing activity-low screen* category. It is possible that more advantaged parents substitute some of their children’s physical activity time with time spent in educational activities—such as reading, singing or taking part in organized lessons [46]. This points to the importance of considering the latter in future research.

There were some differences between the B- and K-cohort in the factors that predicted membership in healthier time-use categories. For example, parental income and SEIFA score were significant predictors for children in the B-cohort, but not for K-cohort children. However, inspection of the point estimates revealed that the direction of association for all covariates was consistent across cohorts. This suggests that differences across cohorts in the few variables which are, and are not, statistically significant are a product of statistical power.

Despite the uniqueness of our findings, several data-driven study limitations must be acknowledged. First, the data used covers the period 2004–2008. Since then, there have been significant developments in children’s opportunities to engage in screen time (e.g., emergence of tablet devices and smartphones) [41, 47]. These new devices may have increased screen time at the expense of other activities in more recent cohorts. These devices may have also changed the type of content that children are viewing during their screen time. As such, it is possible that the results presented here are not generalizable to contemporary children of the target ages. However, our findings are relevant to those children whose trajectories we explored (i.e., children who are now aged 15–19 years old).

Second, the time-use data capture only 2 days in the lives of children each year, and it is possible that these days are not representative of their habitual time-use patterns. This may have introduced measurement error in the analyses, potentially diluting the magnitude of some of the existing associations. Yet, methodological research indicates that time-use diaries capture more valid and reliable information on actual time expenditure than stylised time-use survey questions, and are less invasive and resource intensive than participant observation [48]. Further, the LSAC time-use diaries have been previously used successfully to study both physical activity and screen time [49, 50].

Third, we rely on parent-reported rather than objectively measured (or child-reported) measures of physical activity. Based on methodological studies comparing objective and subjective measures, it is possible that the identified levels of physical activity are over-reported [51]. While it is possible that the parent-reported measures of screen time are also over-reported [41], methods to capture screen time objectively (e.g., wearable cameras) are expensive and difficult to apply in large-scale surveys, particularly for younger children [41, 52].

Fourth, we were unable to derive complete longitudinal trajectories over ages 0–9 years, and instead relied on two separately estimated sets of trajectories for children aged 0–4 years (B-cohort) and 5–9 years (K-cohort). This approach is therefore blind to possible cohort changes in the prevalence, predictors and consequences of the different trajectories. Other study limitations included the need to impute a substantial amount of time-use information to derive longitudinal trajectories, and use of a coarse measure of physical activity that does not distinguish its intensity.

These limitations are nevertheless eclipsed by significant study strengths. These include the use of unique, longitudinal time-use-diary data from a nationally-representative dataset, availability of information on a large number of children, use of a broad range of high-quality, validated measures of children’s HRQoL and social-emotional outcomes, and innovative application of non-parametric growth mixture modelling to identify jointly-determined physical-activity/screen-time trajectories.

## Conclusions

This study generated first-time evidence on the joint physical-activity/screen-time trajectories of infants and young children. Key findings indicate that developmental trajectories characterised by low levels of physical activity and screen time are most common, but those characterised by high levels of physical activity and screen time are associated with the best HRQoL and socio-emotional outcomes. Some groups of children consistently enter time-use trajectories characterised by low levels of physical activity and high levels of screen time, which lead to the worst outcomes. This includes children who are female, from non-English-speaking backgrounds, not living with two biological parents, in affluent households and neighbourhoods, without siblings and whose parents have poorer mental health. Collectively, the evidence suggests that interventions aimed at promoting healthy use of time amongst infants and young children should target these children. The long-term effects of time-use trajectories observed in the data—despite moderate in size—underscore the importance of encouraging children to develop healthy lifestyle habits during the early years. Altogether, these findings have the potential to inform policy development concerning health promotion in infants and young children, stressing the importance of increasing physical activity and reducing screen time. Certain cohorts of at-risk children require more urgent attention.

## Additional files

Additional file 1: **Table S1.** Allocation of pre-determined LSAC time-use categories to physical activity and screen time. (DOCX 24 kb)

Additional file 2: Patterns in and treatment of missing data. (DOCX 23 kb)

Additional file 3: Sensitivity analyses to determine the optimal joint physical-activity/screen-time trajectories according to different methological decisions. (DOCX 16 kb)

Additional file 4: **Table S4.** Average marginal effects for multinomial regression models presented in Table 2. (DOCX 16 kb)

## Data Availability

The datasets generated and/or analysed during the current study are available in the Australian Data Archive repository, https://dataverse.ada.edu.au/dataverse/ada

## Abbreviations

HRQoL: Health-related quality-of-life
LSAC: The Longitudinal Study of Australian Children
SDQ: Strength and difficulties questionnaire
