# Supplementary material for: Joint physical-activity/screen-time trajectories during early childhood: socio-demographic predictors and consequences on health-related quality-of-life and socio-emotional outcomes
Source: Int J Behav Nutr Phys Act. 2019 Jul 8;16:55. doi: 10.1186/s12966-019-0816-3 (PMC6615223; doi:10.1186/s12966-019-0816-3)
Supplement: Supplementary file 1 — Table S1. Allocation of pre-determined LSAC time-use categories to physical activity and screen time. (DOCX 24 kb) [file 12966_2019_816_MOESM1_ESM.docx]

Table S1. Allocation of pre-determined LSAC time-use categories to physical activity and screen time

| ***B-Cohort*** ^c^ | | |
| --- | --- | --- |
| **Wave 1 *(0/1 years)*** | **Wave 2 *(2/3 years)*** | **Wave 3 *(4/5 years)*** |
| *Physical activity*   - Active free play (running, climbing, ball game) - Crawl, climb, swing arms or legs | *Physical activity*   - Active free play (running, climbing, ball game) - Walk for travel or fun - Ride bike, trike, etc. (travel or fun) | *Physical activity*   - Active free play (running, climbing, ball game) - Walk for travel or fun - Ride bike, trike, etc. (travel or fun) |
| *Screen time*   - Watching TV, video or DVD | *Screen time*   - Watching TV, video or DVD - Using computer/computer game | *Screen time*   - Watching TV, video or DVD - Using computer/computer game |
| *Other time uses*   - Bathe/nappy change, dress/hair care - Breastfeeding - Other eating, drinking, being fed - Crying, upset - Destroying things, create mess - Other play, other activities - Visiting people, special event, party - Taken places with adult (shopping) - Not sure - Awake in bed/cot - Looking around, doing nothing - Held, cuddled, comforted soothed - Listening to tapes, CDs, radio, music - Read a story, talked/sung to, sing/talk - Colour/draw, look at book, puzzles - Organized activities/playgroup - Travel in pusher of bike seat - Travel in car - Public transport - Sleeping, napping ^b^ | *Other time uses*   - Quiet free play (e.g., jigsaw, craft, dress-up) - Bathing, dressing, hair care, health care - Eating and drinking, being fed - Crying, upset, tantrum - Destroying things, create mess - Arguing, fighting - Being reprimanded, corrected - Being taught to do chores, read etc - Visiting people, special event, outing - Taken places with adult (shopping) - Not sure - Awake in bed - Doing nothing, bored restless - Held, cuddled, comforted soothed - Listening to tapes, CDs, radio, music - Being read to, told a story, or sung to - Drawing, colouring, looking at book, educational game - Organized lessons/activities - Travel in pusher of bike seat - Travel in car - Public transport - Sleeping, napping ^b^ | *Other time uses*   - Quiet free play (e.g., jigsaw, craft, dress-up) - Bathing, dressing, hair care, health care - Eating and drinking, being fed - Crying, upset, tantrum - Destroying things, create mess - Arguing, fighting - Being reprimanded, corrected - Being taught to do chores, read etc - Visiting people, special event, outing - Taken places with adult (shopping) - Not sure - Awake in bed - Doing nothing, bored restless - Held, cuddled, comforted soothed - Listening to tapes, CDs, radio, music - Being read to, told a story, or sung to - Drawing, colouring, looking at book, educational game - Organized lessons/activities - Travel in pusher of bike seat - Travel in car - Public transport - Sleeping, napping ^b^ |
| ***K-Cohort*** | | |
| **Wave 1 *(4/5 years)*** | **Wave 2 *(6/7 years)*** | **Wave 3 *(8/9 years)*** |
| *Physical activity*   - Walk for travel or fun - Ride bike, trike etc., travel or fun - Other exercise: swim, dance, run about - Other play, other activities * 0.5 ^a^ | *Physical activity*   - Walk for travel or fun - Ride bike, scooter, rollerblades etc. (travel or fun) - Active free play (running, climbing, ball game) - Organized sport, physical activity (swim, dance, Auskick) | *Physical activity*   - Walk for travel or fun - Ride bike, scooter, rollerblades etc. (travel or fun) - Active free play (running, climbing, ball game) - Organized sport, physical activity (swim, dance, Auskick) |
| *Screen time*   - Watching TV, video or DVD - Using computer/computer game | *Screen time*   - Watching TV, video or DVD - Using computer/computer game | *Screen time*   - Watching TV, video or DVD - Using computer/computer game |
| *Other time uses*   - Bathing, dressing, hair care, health care - Eating and drinking, being fed - Crying, upset, tantrum - Destroying things, create mess - Being reprimanded, corrected - Being taught to do chores, read etc. - Visiting people, special event, party - Other play, other activities * 0.5 ^a^ - Taken places with adult (e.g., shopping) - Organized lessons/activities - Not sure - Awake in bed - Doing nothing, bored restless - Held, cuddled, comforted soothed - Listening to tapes, CDs, radio, music - Being read to, told a story, or sung to - Drawing, colouring, looking at book, educational game - Travel in pusher or on bike seat - Travel in car - Public transport - Sleeping, napping ^b^ | *Other time uses*   - Bathing, dressing, hair care, health care - Eating and drinking, - Crying, upset, tantrum - Arguing, fighting, destroying things - Being reprimanded, corrected - Quiet free play (board game, dress-up) - Helping with chores, jobs - Visiting people, special event, party - Taken places with adult (eg shopping) - Organized lesson/activity (music, drama) - Not sure - Awake in bed - Doing nothing, bored restless - Held, cuddled, comforted soothed - Listening to tapes, CDs, radio, music - Being read to, told a story - Reading or looking at book by self - Travel in car - Public transport - Sleeping, napping ^b^ | *Other time uses*   - Bathing, dressing, hair care, health care - Eating and drinking, - Sulking, upset - Arguing, fighting - Being reprimanded, corrected - Quiet free play (board game, craft, dress-up) - Helping with chores, jobs - Visiting people, special event, outing - Taken places with adult (e.g., shopping) - Organized lesson/activity (music, drama) - Not sure - Awake in bed - Doing nothing, bored restless - Being hugged, comforted, helped to calm down - Listening to tapes, CDs, radio, music - Being read to, told a story - Reading or looking at book by self - Travel in car - Public transport - Sleeping, napping ^b^ |

*Notes*: There are issues with the level of precision with which we can identify whether an activity involved physical activity or not (e.g., organized lessons/activities). When it was not clear the activity involved physical activity to a moderate level, we took a conservative approach and excluded such activity from the computation of physical activity time. ^a^ The absence of the category ‘Active free play’ in Wave 1 of the K-cohort (4/5 years), which was available in all other waves, produced marked inconsistencies in physical-activity time relative to both B-Cohort children of the same age (Wave 3) and K-cohort children at ages 6/7 (Wave 2). This issue was successfully resolved by allocating 50% of the time in the ‘Other play, other activities’ category to *Physical Activity* and 50% to *Other uses of time*. ^b^ All missing information between 10pm and 6am is replaced with sleep time. ^c^ Occasionally, several activities were reported to take place within the same 15-minute time slot (e.g., eating and watching TV). When these activities cut across activity types (i.e., physical activity, screen time, other uses of time), time was allocated to each activity type proportional to the number of activities in the 15-minute time slot that belonged in that activity type.
