# Supplementary material for: Joint physical-activity/screen-time trajectories during early childhood: socio-demographic predictors and consequences on health-related quality-of-life and socio-emotional outcomes
Source: Int J Behav Nutr Phys Act. 2019 Jul 8;16:55. doi: 10.1186/s12966-019-0816-3 (PMC6615223; doi:10.1186/s12966-019-0816-3)
Supplement: Supplementary file 2 — Patterns in and treatment of missing data. (DOCX 23 kb) [file 12966_2019_816_MOESM2_ESM.docx]

Additional file 2. Patterns in and treatment of missing data

Table S2.1 Imputed data on physical activity and screen time due to (a) complete wave non-response (due to attrition or non-completion of the time-use diary) and (b) extreme values in the time-use variables

|  | B-cohort  (*n*=4,164) | | K-cohort  (*n*=3,979) | |
| --- | --- | --- | --- | --- |
| Variable | (missing, *n*) | (missing, *%*) | (missing, *n*) | (missing, *%*) |
| Physical activity at wave 1 |  |  |  |  |
| *Missing, n* | 710 | 17.05 | 830 | 20.85 |
| *Top-coded at 99^th^ percentile, n* | 35 | 0.84 | 32 | 0.80 |
| Physical activity at wave 2 |  |  |  |  |
| *Missing, n* | 1,092 | 26.22 | 995 | 25.00 |
| *Top-coded at 99^th^ percentile, n* | 30 | 0.72 | 29 | 0.72 |
| Physical activity at wave 3 |  |  |  |  |
| *Missing, n* | 1,427 | 34.26 | 1,310 | 32.92 |
| *Top-coded at 99^th^ percentile, n* | 27 | 0.64 | 26 | 0.65 |
| Screen time at wave 1 |  |  |  |  |
| *Missing, n* | 710 | 17.05 | 830 | 20.85 |
| *Top-coded at 99^th^ percentile, n* | 35 | 0.84 | 32 | 0.80 |
| Screen time at wave 2 |  |  |  |  |
| *Missing, n* | 1,092 | 26.22 | 995 | 25.00 |
| *Top-coded at 99^th^ percentile, n* | 31 | 0.74 | 30 | 0.75 |
| Screen time at wave 3 |  |  |  |  |
| *Missing, n* | 1,427 | 34.26 | 1,310 | 32.92 |
| *Top-coded at 99^th^ percentile, n* | 28 | 0.67 | 27 | 0.67 |

Note: All missing values were imputed using the copy mean method, as described in the body of the text.

Table S2.2 Missing data in covariates and outcome variables, conditional on participation on the relevant study wave

|  | B-cohort  (*n*^a^=2,737; *n*^b^=3,454) | | K-cohort  (*n*^a^=2,669; *n*^b^=,3149) | |
| --- | --- | --- | --- | --- |
| Variable | (missing, *n*) | (missing, *%*) | (missing, *n*) | (missing, *%*) |
| *Child outcomes (Wave 3)* ^a^ |  |  |  |  |
| Quality of life (PedsQL) |  |  |  |  |
| Total score | 24 | 0.87 | 15 | 0.56 |
| Physical health | 23 | 0.84 | 15 | 0.56 |
| Social health | 35 | 1.27 | 17 | 0.63 |
| Emotional health | 25 | 0.91 | 15 | 0.56 |
| Socio-emotional functioning (SDQ) |  |  |  |  |
| Total score | 20 | 0.73 | 17 | 0.63 |
| Hyperactivity | 20 | 0.73 | 17 | 0.63 |
| Peer problems | 19 | 0.69 | 16 | 0.59 |
| Conduct problems | 19 | 0.69 | 16 | 0.59 |
| Emotional problems | 19 | 0.69 | 15 | 0.56 |
| *Predictors/covariates (Wave 1)*  ^b^ |  |  |  |  |
| Female | 0 | 0 | 0 | 0 |
| Indigenous | 0 | 0 | 1 | 0.03 |
| Socio-Economic Index for Areas | 0 | 0 | 0 | 0 |
| Low birth weight | 12 | 0.34 | 32 | 1.01 |
| Main caregiver mental health | 42 | 1.21 | 61 | 1.93 |
| English as first language | 2 | 0.05 | 0 | 0 |
| Siblings | 0 | 0 | 0 | 0 |
| Two biological parents | 0 | 0 | 0 | 0 |
| Parental income | 0 | 0 | 0 | 0 |

Note: ^a^ Of all children who participated in study Wave 3 and completed the time-use diary. ^b^ Of all children who participated in study Wave 1 and completed the time-use diary.
