# Supplementary material for: Joint physical-activity/screen-time trajectories during early childhood: socio-demographic predictors and consequences on health-related quality-of-life and socio-emotional outcomes
Source: Int J Behav Nutr Phys Act. 2019 Jul 8;16:55. doi: 10.1186/s12966-019-0816-3 (PMC6615223; doi:10.1186/s12966-019-0816-3)
Supplement: Supplementary file 3 — Sensitivity analyses to determine the optimal joint physical-activity/screen-time trajectories according to different methological decisions. (DOCX 16 kb) [file 12966_2019_816_MOESM3_ESM.docx]

Additional file: 3. Sensitivity analyses to determine the optimal joint physical-activity/screen-time trajectories according to different methological decisions.

A series of sensitivity analyses were conducted to test the robustness of the results to different methodological decisions. First, the centroids used to generate the latent trajectories were derived based on sample *median* scores, which are less likely to be affected by outliers than sample *mean* scores. Using this approach, three-cluster solutions for joint physical-activity/screen-time trajectories were identified for both B-cohort children (*Low activity-low screen*, 48.7%; *Increasing activity-low screen*, 27.4%; *Low activity-increasing screen*, 24%; n= 4,164) and K-cohort children (*Low activity-low screen*, 48%; *Increasing activity-low screen*, 28%; *Low activity-increasing screen*, 24%; n=3,979). These were very similar to those reported in the main text.

Second, we repeated all analyses using only complete cases, i.e., children for which time-use diaries were available for all three study waves (*n*=1,938 for B-cohort children & *n*=1,838 for K-cohort children)*.* Using this approach, three-cluster solutions for joint physical-activity/screen-time trajectories were also identified for both B-cohort children (*Low activity-low screen*, 49.4%; *Increasing activity-low screen*, 26.7%; *Low activity-increasing screen*, 22.4%) and K-cohort children (*Low activity-low screen*, 50%; *Increasing activity-low screen*, 27.6%; *Low activity-increasing screen*). These were also very similar to those reported in the main text.

Third, longitudinal weights for both B-cohort and K-cohort were used in the identification of the latent trajectories. Using this approach, three-cluster solutions for joint physical-activity/screen-time trajectories were also identified for both B-cohort children (*Low activity-low screen*, 49.3%; *Increasing activity-low screen*, 26.7%; *Low activity-increasing screen*, 24%) and K-cohort children (*Low activity-low screen*, 49.7%; *Increasing activity-low screen*, 27.6%; *Low activity-increasing screen*, 22.6%). Again, these were very similar to those reported in the main text. Although *a priori* the use of longitudinal weights could be deemed as the preferred course of action, longitudinal weights in the LSAC data were only available for children observed in all three time periods. This reduced the number of cases for analysis to 2,681 and 2,614 for the B-cohort and K-cohort, respectively. It also introduced selectivity issues.
