# Supplementary material for: Joint physical-activity/screen-time trajectories during early childhood: socio-demographic predictors and consequences on health-related quality-of-life and socio-emotional outcomes
Source: Int J Behav Nutr Phys Act. 2019 Jul 8;16:55. doi: 10.1186/s12966-019-0816-3 (PMC6615223; doi:10.1186/s12966-019-0816-3)
Supplement: Supplementary file 4 — Table S4. Average marginal effects for multinomial regression models presented in Table 2. (DOCX 16 kb) [file 12966_2019_816_MOESM4_ESM.docx]

| Table S4. Average marginal effects for multinomial regression models presented in Table 2 | | | |
| --- | --- | --- | --- |
|  | *Low activity-*  *low screen* | *Increasing Activity-low screen* | *Low activity-increasing screen* |
| **B-cohort (0-5 years)** |  |  |  |
| Female, *yes* | 0.135^***^ | -0.064^***^ | -0.071^***^ |
| Indigenous, *yes* | -0.071 | -0.052 | 0.123 |
| SEIFA (0-14) | 0.056^***^ | -0.030^**^ | -0.026^*^ |
| Low birth weight, *yes* | 0.064 | -0.037 | -0.027 |
| Main caregiver mental health (0-5) | -0.039^*^ | 0.039^*^ | 0.000 |
| E English as first language, *yes* | 0.006 | 0.062^*^ | -0.069^*^ |
| Siblings, *yes* | -0.076^***^ | 0.075^***^ | 0.001 |
| T Two biological parents, *yes* | -0.101^*^ | 0.087^**^ | 0.014 |
| Weekly parental income (in AU$1,000) | 0.036^***^ | -0.014 | -0.022^*^ |
| **K-cohort (4-9 years)** | | | |
| Female, *yes* | 0.181^***^ | -0.085^***^ | -0.096^***^ |
| Indigenous, *yes* | -0.077 | -0.015 | 0.092 |
| SEIFA (0-14) | 0.028^*^ | 0.003 | -0.032^**^ |
| Low birth weight, *yes* | -0.022 | 0.047 | -0.025 |
| Main caregiver mental health (0-5) | -0.004 | 0.039^*^ | -0.035^*^ |
| English as first language, *yes* | -0.121^***^ | 0.106^***^ | 0.016 |
| Siblings, *yes* | -0.047^*^ | 0.055^**^ | -0.008 |
| Two biological parents, *yes* | -0.072^*^ | 0.066^*^ | 0.006 |
| Weekly parental income (in AU$1,000) | 0.009 | -0.002 | -0.006 |
| Notes: LSAC data, Waves 1-3. SEIFA: Socio-Economic Index for Areas. ^*^ *p*<0.05, ^**^ *p*<0.01, ^***^ *p*<0.001. | | | |
